# Metastasis of prostate cancer to breast: a case report

**DOI:** 10.3389/fonc.2025.1580441

**Published:** 2025-05-16

**Authors:** Sirui Zhang, Wenting Peng, Lin Cheng, Bei Pei, Xiaoli Zhou, Mengjie Wang, Ling Jiang, Chao Lu, Lingyun Xu

**Affiliations:** ^1^ Department of Breast Surgery, The Third Affiliated Hospital of Nanjing Medical University, Changzhou Second People’s Hospital, Changzhou, Jiangsu, China; ^2^ Department of Pathology, The Third Affiliated Hospital of Nanjing Medical University, Changzhou Second People’s Hospital, Changzhou, Jiangsu, China; ^3^ Department of Oncology, The Third Affiliated Hospital of Nanjing Medical University, Changzhou Second People’s Hospital, Changzhou, Jiangsu, China; ^4^ Department of Urology Surgery, The Third Affiliated Hospital of Nanjing Medical University, Changzhou Second People’s Hospital, Changzhou, Jiangsu, China

**Keywords:** prostate cancer, breast metastasis, male breast cancer, BRCA mutations, androgen receptor, differential diagnosis

## Abstract

We describe a 68-year-old man who had prostate cancer (PCa) diagnosed with breast metastasis, presenting as a breast mass. Seven years ago, the patient had PCa with bone metastasis and had been systematically receiving endocrine therapy for prostate cancer and osteoprotective treatment since the diagnosis. In October 2023, the patient visited our hospital due to bilateral breast masses for four days. Enlarged supraclavicular lymph nodes were detected, while multiple solid space-occupying lesions were observed in bilateral mammary tissues, classified as Breast imaging reporting and data system (BI-RADS) 4C. Subsequently, he underwent lumpectomy and was diagnosed with metastatic prostatic acinar adenocarcinoma.

## Introduction

Male breast cancer (MBC) is a rare disease, with an incidence rate of 0.5% to 1% compared with female breast cancer (1). Meanwhile, previous studies have shown that breast metastatic cancer is very rare, accounting for only 0.3% to 2.7% of all breast malignancies (2). As a result, the incidence of metastatic male breast cancer is extremely rare. Owing its rarity, the diagnosis and treatment of male breast cancer primarily follow the same guidelines as those for female breast cancer. However, there are differences in the management of male and female breast cancer, differential diagnosis is also necessary to distinguish between metastatic and primary male breast cancer. Here, we present a rare case of breast metastasis from prostate cancer.

## Case presentation

**Figure 1:**
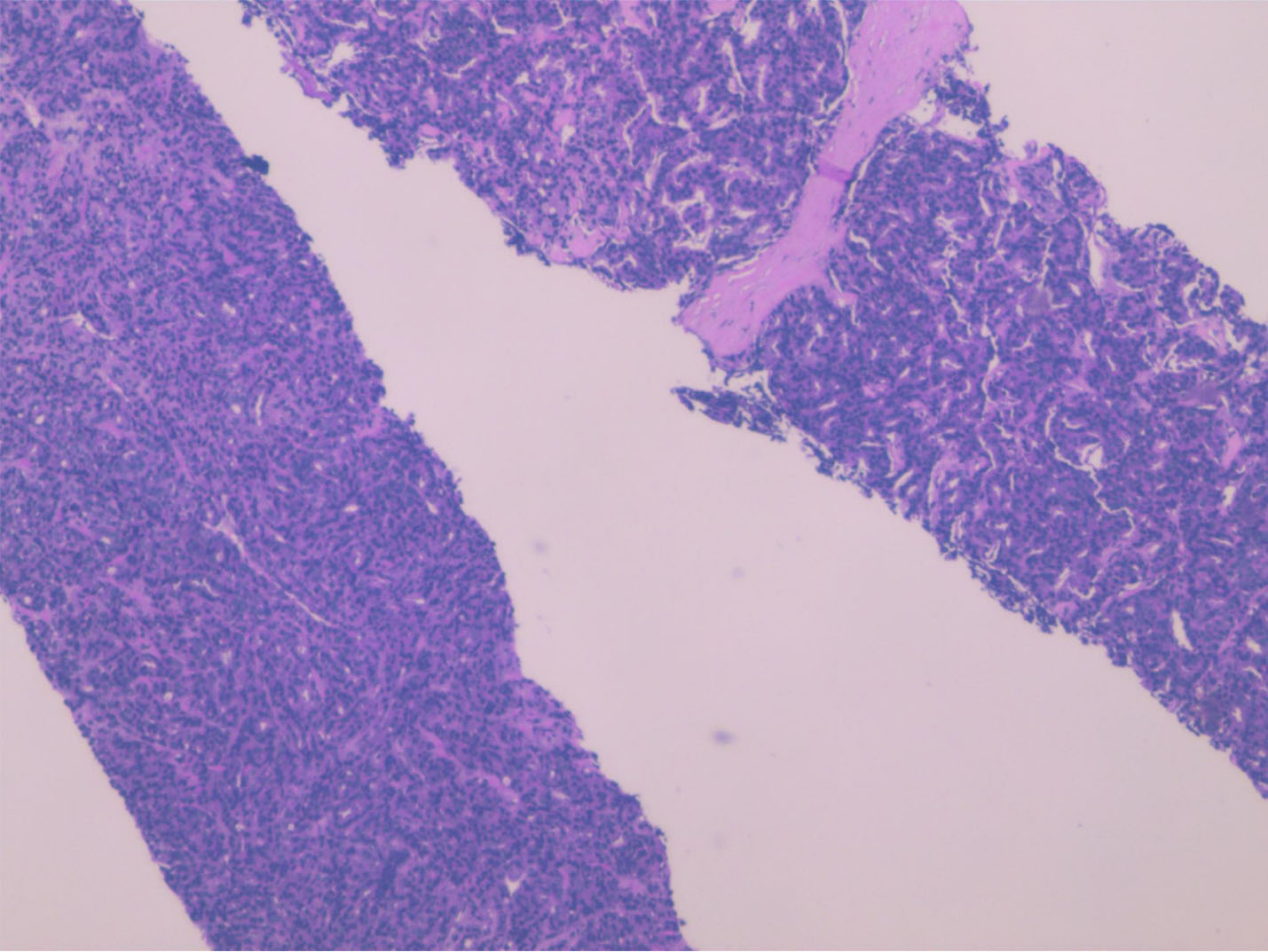
Histopathological examination of the ultrasound-guided prostate biopsy demonstrated Left-sided prostatic adenocarcinoma (Gleason score of 5 + 4).

A 68-year-old male patient suffered from neck and back pain presented to our hospital seven years ago. Magnetic resonance imaging (MRI) showed pathologic fracture at T5, and abnormal signals at the left anterior edge of T6 vertebrae, indicating either tuberculosis or bone metastasis. Notably, his prostate-specific antigen (PSA) level was measured at 48.49ng/ml, and whole-body bone imaging suggested the presence of metastatic bone tumor. Further pelvic MRI revealed abnormal signal shadows in the left peripheral gland of the prostate. To obtain a definitive diagnosis, an ultrasound-guided prostate biopsy was performed. Pathological examination showed prostate cancer on the left side, with a Gleason score of 5 **+** 4 (**Figure 1**), while the right side exhibited benign hyperplasia. Consequently, the patient was diagnosed with prostate cancer with bone metastasis and was treated with castration + endocrine therapy (Bicalutamide + Goserelin acetate) and osteoprotective treatment (Zoledronic acid).

In 2020, the patient’s computed tomography (CT) and MRI scans revealed multiple metastases in the spine at levels C7, T3-5, T7, T11-12, and L2-4, with associated soft tissue mass formation. There was evidence of a pathologic fracture of T4 vertebral body. Suspected metastasis to the left adrenal gland was noted, along with multiple areas of increased bone mineral density in the ribs on the left side with local soft tissue foci. Additionally, metastatic malignant foci in the cervical and thoracic vertebral regions was identified. Multiple increases in bone mineral density were observed in the cervicothoracolumbar vertebrae, right sacroiliac joints, and left acetabulum, indicating a high likelihood of metastasis. The patient’s condition progressed to metastatic castration-resistant prostate cancer (mCRPC). Consequently, the treatment was adjusted to “Abiraterone + Prednisone” in November 2020, and “Zoledronic acid” osteoprotective treatment was added from that time onwards.

**Figure 2:**
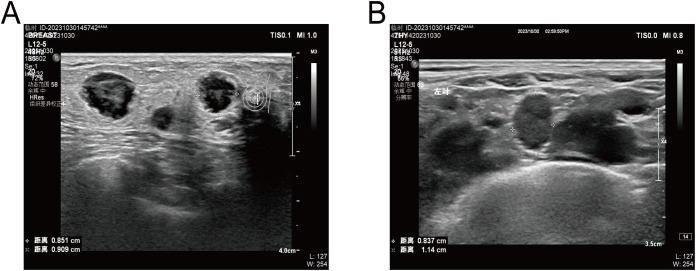
**(A)**, Ultrasound showed several hypoechoic masses with blurred margins and irregular morphology. **(B)**, Ultrasound showed enlarged lymph nodes in his left supraclavicular region.

In 2023, MRI revealed multiple metastases in the cervicothoracic, lumbar, and sacral vertebrae, as well as in the adnexal bones. Abdominal and pulmonary CT scans suggested possible metastases of pelvic and abdominal lymph nodes. Additionally, the PSA level was measured at over 100ng/ml. In October, the patient visited our hospital due to bilateral breast masses that had been presented for four days. Physical examination revealed multiple hard lumps in his breasts. Ultrasound imaging indicated the presence of multiple bilateral breast nodules. Unlike benign masses, which typically grow horizontally with well-defined borders and regular morphology, the patient’s nodules exhibited vertical growth with blurred margins, irregular morphology, and abundant blood flow signals, classifying them as BI-RADS 4C. Additionally, enlarged lymph nodes were detected in his left supraclavicular region, while his right supraclavicular and bilateral axillary lymph nodes did not show significant enlargement (**Figure 2**). Given the patient’s history of prostate cancer and his receipt of standardized hormone therapy, we suspected that hormonal changes might have led to gynecomastia, potentially creating a conducive environment for the development of primary breast cancer. However, since gynecomastia is relatively uncommon in cases of male primary breast cancer, we decided to perform a surgical excision of the masses. This procedure will allow us to further clarify the nature of the disease through pathological examination and also halt its progression.

**Figure 3:**
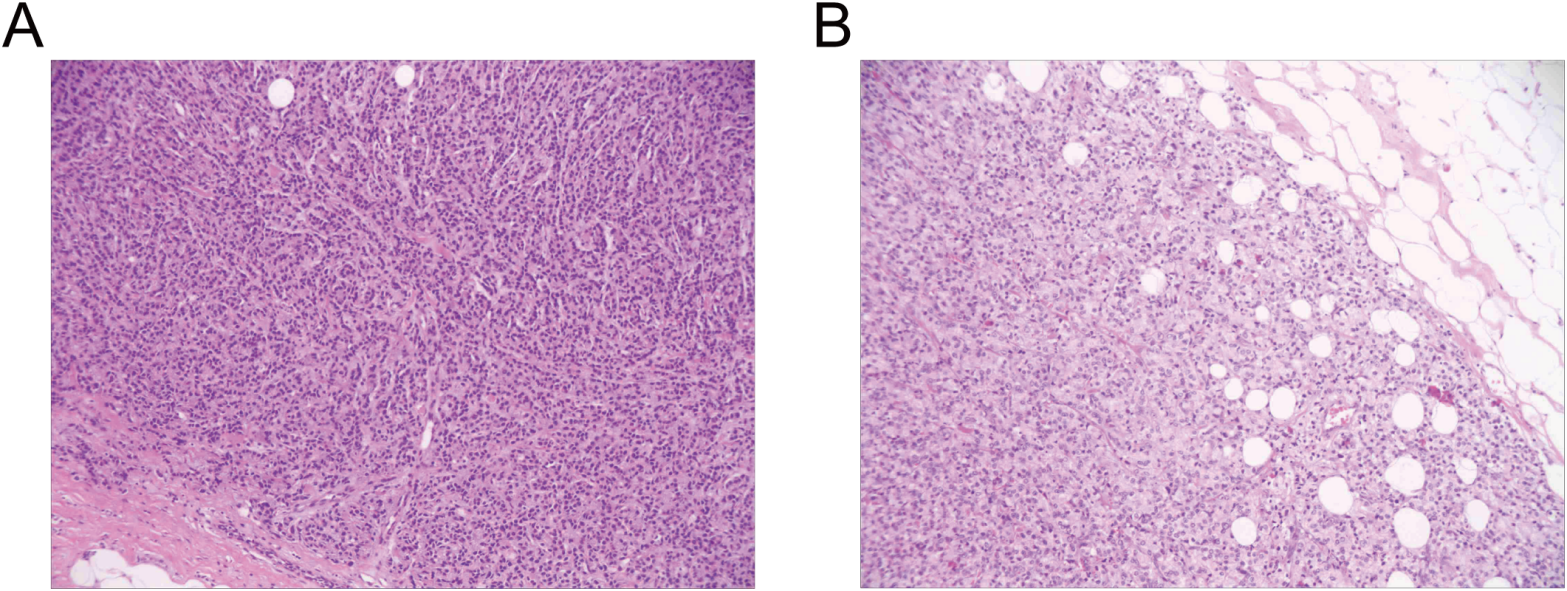
Routine pathology examination of the excised breast lesion demonstrated metastatic infiltration by prostatic adenocarcinoma. **(A)**, Left breast mass: metastatic prostatic acinar adenocarcinoma. **(B)**, Right breast mass: metastatic prostatic acinar adenocarcinoma.

**Figure 4:**
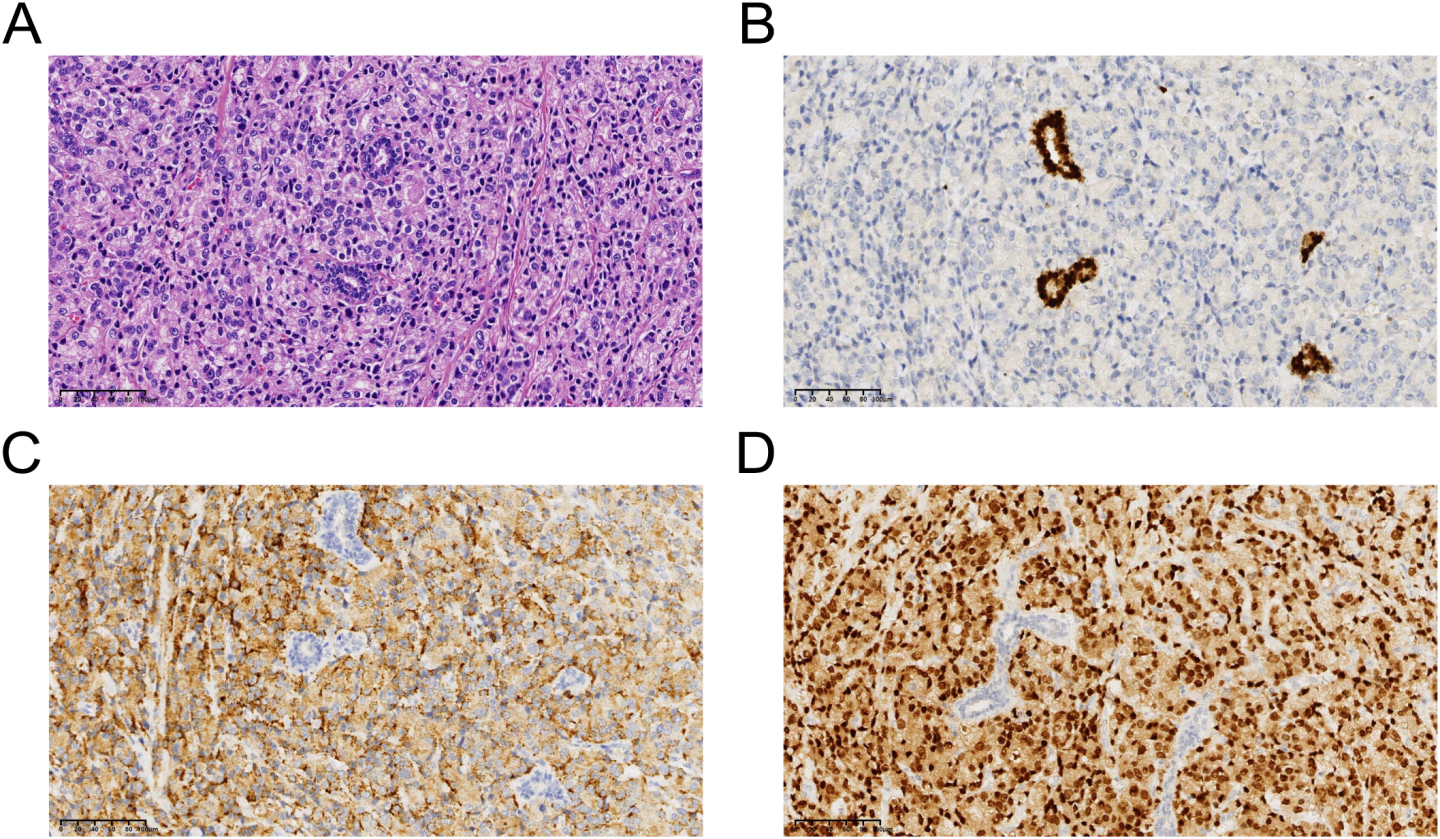
**(A)**, Representive photomicrographs of the metastatic prostatic acinar adenocarcinoma (hematoxylin-eosin staining: magnification, ×40). **(B)**, GATA Binding Protein 3: negative. **(C)**, Prosaposin: positive. **(D)**, NK3 Homeobox 1: positive. .

In November, the patient underwent a lumpectomy at the Department of Breast Surgery in our hospital. Interestingly, frozen section pathology suggested that the masses were invasive or metastatic cancer. We further conducted routine pathology and immunohistochemical analysis of the masses, confirming metastatic prostatic acinar adenocarcinoma in both mammary tissues (**Figure 3**). Immunohistochemistry (IHC) staining showed negative expression of GATA Binding Protein 3 (GATA-3) and positive expression of ETS-related gene (ERG), Prosaposin (PSAP), and NK3 Homeobox 1 (NKX3.1) (**Figure 4**). Genetic testing revealed a Breast cancer susceptibility gene 2 (BRCA2) mutation, androgen receptor(AR) gene amplification, and microsatellite stability (MSS). Based on these findings, we recommend a combination of targeted therapy, chemotherapy, and osteoprotective treatment for the patient. Besides, the patient is currently receiving anti-androgen therapy (Triptorelin) and osteoprotective treatment due to elevated testosterone levels. Through regular follow-up, the patient’s condition remains stable up to now, and we will continue to track his disease progression closely.

## Discussion

In the present case, prostate cancer metastasized to the bilateral breasts, which were rare metastatic sites of prostate cancer. Bones are the most common sites for prostate cancer, followed by the lungs, liver, and brain, with metastasis to other sites being comparatively rare (3). Carcinoma of the prostate presenting with breast metastasis is rarely reported in the literature. It has been documented that the increased secretion of luteinizing hormone (LH) during the initial phase of hormonal therapy for prostate cancer can lead to an imbalance in estrogen levels, predisposing to breast cancer metastasis (4). In the past, estrogen therapy was considered the main treatment for prostate cancer, but it has also been implicated as a cause of breast cancer metastasis (5, 6). In this report, we utilized combined androgen blockade(CAB) therapy with “ Bicalutamide + Goserelin acetate”, which can increase testosterone levels by blocking negative feedback to LH, thereby increasing estrogen levels through aromatization of testosterone. It has been previously documented that estrogen causes proliferative changes in the breast, with development of both ducts and periductal stroma (7).

Breast cancer susceptibility gene1/2(BRCA1/2) are tumor suppressor genes involved in DNA repair and maintenance of genomic stability (8). Men with BRCA1/2 mutations have an increased risk of developing breast cancer, prostate cancer, pancreatic cancer, and other malignancies (8–11). Notably, among individuals with BRCA2 mutations, prostate cancer is the most frequently diagnosed cancer, followed by breast cancer (8). BRCA-mutated prostate cancer is often more aggressive (Gleason scores ≥8) and prone to early metastasis (12). This patient presented with widespread metastases and high Gleason scores at diagnosis, and we speculate that BRCA2 mutation may also contribute to a higher risk of breast metastasis. Additionally, AR acts as a promoter in prostate cancer. Meanwhile, AR plays an indirect role in promoting tumorigenesis and progression by modulating immunity (suppressing the activity and stemness of tumor-infiltrating CD8^+^T cells) (13). The presence of AR gene amplification in the metastatic lesion could be a driving factor behind the metastasis and the tumor may exhibit poor responsiveness to immunotherapy.

Meanwhile, the prognosis and treatment of primary male breast cancer and breast metastases vary significantly. The traditional treatment for primary male breast cancer involves wide local excision or mastectomy with axillary node clearance, while breast metastases are typically managed with systemic chemotherapy. Therefore, it is crucial to differentiate between primary and secondary carcinoma (14). Metastatic PCa is often characterized by metastatic foci with elevated serum PSA levels (15). On the other hand, histopathologic examination of breast lumps, including PSA and PSAP staining on immunohistochemistry, is also helpful in distinguishing the nature of the pathology (16, 17). In this patient’s case, the PSA level increased to over 100 ng/ml when breast lesions were detected by ultrasound. Furthermore, IHC staining demonstrated positive expression of PSAP.

Breast metastatic cancer is very rare, accounting for only 0.3% to 2.7% of breast malignancies (2). This rarity may be related to the fact that breasts contain a large amount of fibrous tissue and have relatively poor blood supply (18). Most breast metastases originate from the contralateral breast, and other primary tumors that tend to metastasize to the breast include melanoma, lymphoma, ovarian cancer, lung cancer, neuroendocrine tumors, and sarcoma. Breast metastases often lack distinctive features on imaging so far. They typically appear as round or elliptical masses with well-defined borders, making it challenging to differentiate them from primary benign or malignant breast tumors. However, unlike primary carcinomas, breast metastases generally do not have spiculated margins, skin retraction, or nipple retraction (19). Additionally, MRI of breast metastatic tumors often shows signals on T1-weighted imaging (T1WI) and T2-weighted imaging (T2WI), with rapid and obvious homogeneous enhancement in the early stage of enhancement scanning. Platform-type or outflow-type time-signal curves are frequently observed (20).

## Conclusions

Breast metastasis originating from prostate cancer is relatively uncommon, and its clinical symptoms and imaging characteristics often lack distinctive features. Consequently, there is a significant risk of misdiagnosis or underdiagnosis, which can adversely affect patient treatment and prognosis. In addition, the precise mechanism underlying breast metastasis from prostate cancer remains unclear. Therefore it is important to distinguish metastatic lesions of unknown origin through immunohistochemical staining to accurately identify the primary malignant tumor. This is important as it guides the treatment approach and prognosis. In this case, we emphasize the need to differentiate a male breast mass from a breast metastasis when encountering it.

## Data Availability

The original contributions presented in the study are included in the article/supplementary material. Further inquiries can be directed to the corresponding authors.
